# Spoilage-Associated Bacteria in Fresh Cow Cheeses: Diversity, Spoilage-Related Changes, Quantification and Identification Approaches—A Scoping Review

**DOI:** 10.3390/microorganisms14091936

**Published:** 2026-09-02

**Authors:** Miguel Anchundia, Stalin Santacruz

**Affiliations:** 1 Food Engineering Program, Universidad Politécnica Estatal del Carchi, Tulcán 040102, Ecuador; 2 School of Agroindustrial Engineering, Universidad Laica Eloy Alfaro de Manabí, Manta 130222, Ecuador; stalin.santacruz@uleam.edu.ec

**Keywords:** fresh cow cheese, spoilage microbiota, *Pseudomonas* spp., dairy microbiology, next-generation sequencing, shelf life

## Abstract

Fresh cheeses are highly perishable dairy products due to their high moisture content, pH, and other intrinsic characteristics that support microbial growth. This scoping review synthesized the available evidence on spoilage-associated bacteria in fresh cow cheeses, the physicochemical and sensory changes associated with spoilage, and the methods for their identification and quantification. The review was conducted in accordance with the PRISMA-ScR guidelines and registered in the Open Science Framework (OSF). Literature searches were performed in PubMed/MEDLINE, Scopus, SciELO, and Google Scholar through 2 April 2026. Of 7379 records identified, 30 studies met the inclusion criteria. Across the reviewed studies, *Pseudomonas* spp. emerged as the predominant spoilage-associated bacteria, followed by members of the *Enterobacteriaceae*, lactic acid bacteria (LAB), and spore-forming bacteria. Total viable count was the microbiological indicator most frequently used to monitor spoilage progression. Microbial spoilage was consistently associated with acidification, proteolysis, lipolysis, discoloration, gas production, texture deterioration, and reduced sensory acceptability. These findings highlight the central role of psychrotrophic bacteria in the spoilage of fresh cow cheeses and emphasize the importance of microbiological monitoring and spoilage-control strategies throughout production and storage.

## 1. Introduction

Cheese is defined as a soft, semi-hard, hard, or extra-hard product, either ripened or unripened, which may be coated, and in which the ratio of whey proteins to casein does not exceed that present in milk [1]. Cheese diversity is influenced by multiple factors, including milk origin, milk blends, shape, size, texture, aroma, and manufacturing practices. In addition, variations arise from technological processes such as coagulation, curd cutting, whey drainage, washing, salting, and the incorporation of additives, including spices and colorants [2,3].

Unripened cow cheese, commonly referred to as “fresh” or “white cheese,” is characterized by a soft texture and high moisture content and is typically produced from whole or partially skimmed milk. These cheeses are coagulated using enzymes and/or organic acids, generally without starter cultures [4], and may be made from raw or pasteurized milk, depending on local regulations and processing practices. This category includes products such as Stracciatella, Burrata, Minas Frescal, and traditional Latin American cheeses (e.g., Amasado and Manaba), as well as pasta filata varieties such as Mozzarella, Fior di latte, and Oaxaca cheese [5,6].

Intrinsic characteristics of fresh cheese, including pH, water activity, sodium chloride content, moisture, protein, fat, and total carbohydrates, provide favorable conditions for the growth of spoilage microorganisms. These properties make it particularly susceptible to microbial spoilage, even under refrigerated storage, resulting in rapid quality deterioration and a significantly reduced shelf life. In products such as Queso Amasado, shelf life may range from less than one month to as little as fourteen days, depending on the level of microbial contamination and storage conditions [7,8].

Microbial spoilage results in chemical, biochemical, and physical changes driven by microbial activity and enzymatic processes, leading to significant alterations in sensory attributes such as color, taste, odor, flavor, and texture. It also alters the nutritional composition of cheese, including its water, protein, carbohydrate, and lipid contents, thereby reducing the product’s commercial value [9,10]. Consequently, spoilage contributes to food loss, as products are discarded as waste, negatively impacting food and nutritional security, the environment, and the economy [11].

Spoilage-associated bacteria in fresh cheese include psychrotrophic and Gram-negative bacteria, coliforms, *Escherichia coli*, lactic acid bacteria, and proteolytic species such as *Bacillus* spp. (*B. stearothermophilus*, *B. licheniformis*, *B. coagulans*, *B. cereus*, *B. subtilis*, and *B. circulans*), as well as lipolytic bacteria belonging to genera such as *Pseudomonas* and *Enterobacter*, among others. Species of the genus *Pseudomonas* are widely recognized as major spoilage organisms due to their ability to grow at low temperatures, form biofilms in post-processing environments, and produce extracellular enzymes, such as proteases and lipases [12,13]. These enzymatic activities contribute to protein and lipid degradation, leading to defects such as off-flavors, slime formation, texture degradation, and discoloration, including the characteristic blue spoilage phenotype [14,15].

The study of spoilage in cow fresh cheeses has traditionally relied on culture-dependent methods, including microbial cultivation, isolation, and identification. Many microorganisms remain difficult to recover under routine culture conditions [16,17]. To overcome these limitations, culture-independent methods based on molecular techniques, such as polymerase chain reaction (PCR) and next-generation sequencing, are increasingly employed. These approaches enable the identification and characterization of microbial communities, allowing more precise descriptions of microbial diversity and dynamics, as well as a more comprehensive characterization of the cheese microbiota, particularly with respect to spoilage-associated microorganisms [18].

Understanding the spoilage microbiota of fresh cow cheeses is essential for controlling microbial proliferation throughout the production, commercialization, and distribution chain. It also supports the development of effective preservation, sanitation, and packaging strategies, thereby facilitating market expansion and improving the availability of safe, high-quality products. In addition, these approaches contribute to waste reduction and promote more sustainable fresh cheese production. Despite the growing body of research on spoilage microorganisms in individual fresh cheese varieties, information on spoilage-associated bacterial communities, their impact on product quality, and the methodologies used to characterize them remain fragmented across studies. Therefore, this scoping review aimed to synthesize the diversity of spoilage-associated bacterial communities in fresh cheeses, examine the methodologies used to identify and quantify them, and evaluate their impact on physicochemical properties, sensory characteristics, and shelf life.

## 2. Research Methodology

### 2.1. Protocol and Registration

The protocol for this scoping review was established a priori and developed in accordance with the Preferred Reporting Items for Systematic Reviews extension for Scoping Reviews (PRISMA-ScR) guidelines [19]. The protocol was registered in the Open Science Framework (OSF) under the identifier osf-registrations-y8rvp-v1.

To guide the design and structure of this scoping review, the research question was formulated using the PCC framework (Population, Concept, and Context) as described below:

P (Population): Fresh cow cheeses intended for human consumption, including high-moisture, unripened, soft, white, or short shelf-life cheeses.

C (Concept): Spoilage-associated bacterial communities, the physicochemical, sensory, and shelf-life changes associated with spoilage, and the methodologies used for their identification and quantification.

C (Context): Production, storage, commercialization, and refrigerated shelf-life conditions of fresh cow cheeses in industrial, artisanal, or retail environments.

### 2.2. Focused Research Question (PCC)

What evidence is available on spoilage-associated bacterial communities in fresh cow cheeses, the physicochemical and sensory changes associated with spoilage, and the methodologies used to identify and quantify them throughout production, storage, commercialization, and refrigerated shelf life?

### 2.3. Search Strategy

A comprehensive literature search was conducted in PubMed/MEDLINE, Scopus, SciELO, and Google Scholar through 2 April 2026. The general search strategy was based on three conceptual domains, cheese matrix, microbial spoilage, and bacteria or microbiota.

(“cheese” OR “fresh cheese” OR “soft cheese” OR “unripened cheese” OR queso OR “queso fresco” OR “queso blando”) AND (spoilage OR deteriorat* OR “food spoilage” OR “microbial spoilage” OR deterioration OR deterioro OR alteracion OR descomposicion) AND (bacter* OR microbi* OR microbiota OR microflora OR bacteria OR bacterias OR microbiología OR microbiology).

Database-specific search strategies were adapted to the syntax, indexing characteristics, and coverage of each source and are detailed in Table 1. For the SciELO search, complementary English and Spanish terms were retained to enhance retrieval of regional literature, particularly studies from Latin America, where fresh cheeses are widely produced and investigated. Similarly, bilingual queries were maintained in Google Scholar because of its broad academic coverage and complementary role in identifying additional regional records. These terms were used to improve retrieval sensitivity and did not constitute a language-based eligibility criterion.

For Google Scholar, a predefined stopping rule specified in the registered review protocol was applied; the first 100 records retrieved for each search equation were screened, whereas all available records were screened when fewer than 100 were retrieved. This approach is consistent with guidance for search engine searching, which recommends restricting screening to a predetermined number of results, such as the first 100 [20].

### 2.4. Articles Selection

**Figure 1:**
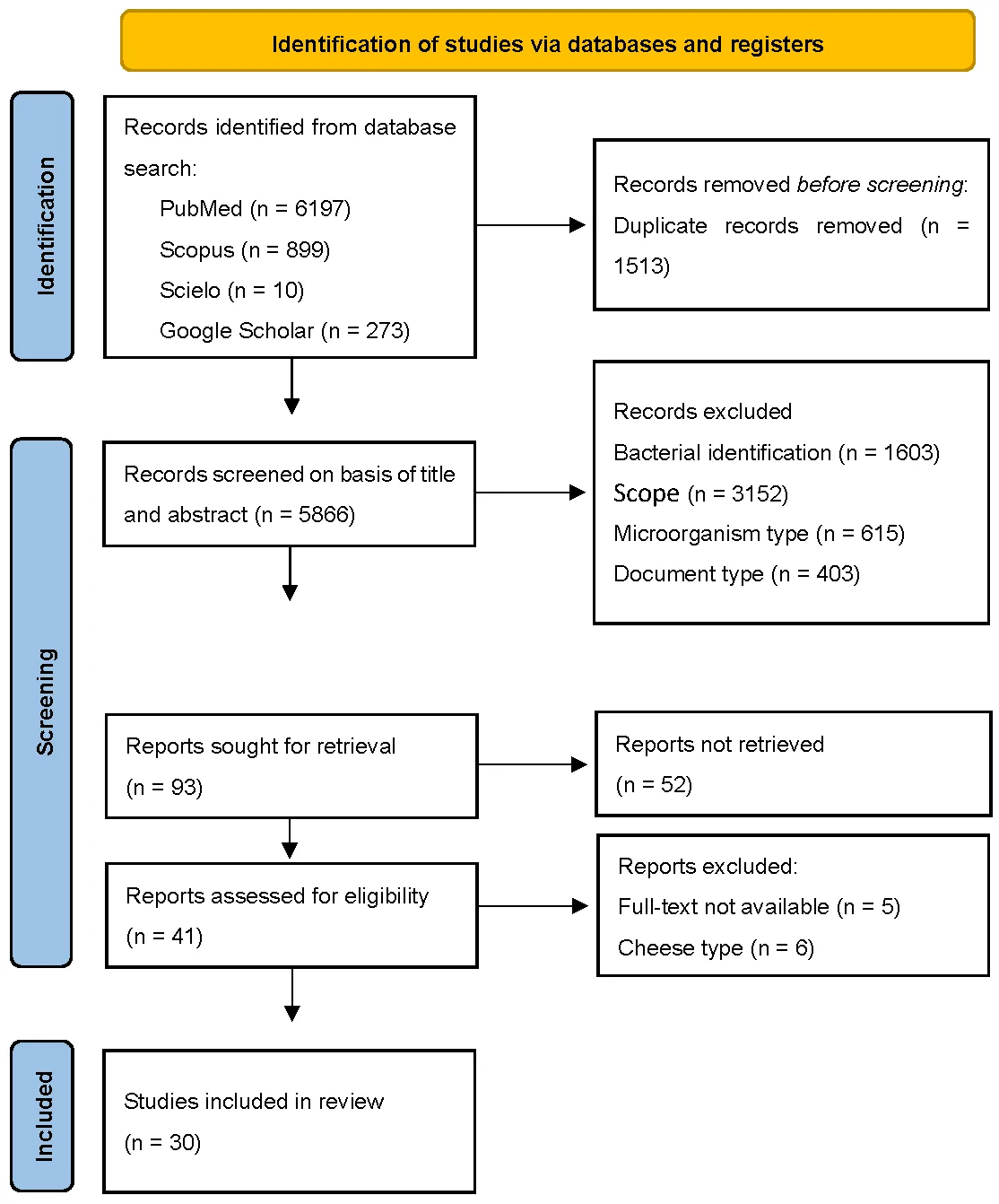
PRISMA flow diagram showing the stages of the study selection process.

Following application of the search strategy, we retrieved 7379 records meeting the criteria presented in Table 2. The study selection process was conducted in accordance with the flow diagram shown in Figure 1. The literature search was performed independently by two reviewers.

Retrieved records were exported to Zotero (Version 9.0) for duplicate removal and reference management.

Subsequently, the authors (M.A. and S.S.) conducted the screening phase by evaluating documents by reference type and reviewing titles and abstracts against predefined eligibility criteria. The selection phase was carried out through full-text assessment, with reasons for exclusion formally documented in accordance with the criteria described above and in line with the PRISMA guidelines.

Microorganisms with recognized pathogenic or hygiene-indicator significance were considered only when the included study also reported a spoilage-associated role; studies addressing these microorganisms exclusively in relation to food safety or hygiene, without spoilage-related outcomes, were excluded.

Both phases were conducted independently by the two authors, who assessed titles and abstracts to minimize bias. Full-text evaluation was performed according to the established eligibility criteria, and any discrepancies between reviewers were resolved by consensus among the two authors.

### 2.5. Data Extraction Process

Data from each eligible study were extracted and synthesized using a predefined structured extraction matrix that included study title, cheese type, spoilage-associated bacteria or bacterial groups reported, main methodological approach, main spoilage-related finding, among others (Supplementary File S2—Scoping Review Spoilage Microorganisms in Fresh Cow Cheese.xlsx). Given the heterogeneity among studies, microbial groups and spoilage outcomes were categorized according to the terminology and classifications reported by the original authors.

This predefined structured extraction matrix served as the basis for constructing the summary tables and narrative synthesis.

Both researchers (M.A. and S.S.) independently extracted data using a predefined structured extraction matrix aligned with the review objectives. Discrepancies were resolved by jointly re-examining the corresponding full-text article, verifying the extracted information against the predefined extraction fields, and discussing any differences until consensus was reached between both researchers.

### 2.6. Critical Appraisal of Sources of Evidence

Consistent with the objectives of scoping review methodology and PRISMA-ScR guidelines, no formal critical appraisal or risk-of-bias assessment of the included studies was performed, as the purpose of this review was to comprehensively map and synthesize the available evidence on spoilage-associated bacteria in fresh cow cheeses rather than evaluate methodological quality.

## 3. Results

### 3.1. General Description of the Selected Articles

The search strategy identified 7379 records, of which 1513 duplicates were removed. Following title and abstract screening, 93 articles were assessed for eligibility through full-text review. Fifty-two records were excluded, primarily because they did not meet the predefined inclusion criteria. Ultimately, 30 studies were retained for qualitative synthesis (Figure 1 and Supplementary File S1—Optimized_Matrix_Q1 Journal.xlsx).

The characteristics of the included studies are summarized in Table 3. Among the studies that met predefined eligibility criteria, publication years ranged from 1992 to 2025, and study settings spanned countries in Europe and Latin America. The analyzed products consisted primarily of high-moisture fresh cheeses, with Mozzarella [21,22,23,24,25,26,27,28,29,30] and soft, white, fresh or fresco cheeses [13,15,31,32,33,34,35,36,37] being the most frequently reported (33.33%, n =10 and 30%, n = 9 respectively), followed by Fior di latte (16.67%, n = 5) [38,39,40,41,42], Minas cheese (6.67%, n = 2) [43,44] and Oaxaca, Panela and Cottage (3.33%, n =1) [12].

### 3.2. Major Spoilage Bacteria in Fresh Cheeses

Among the studies reporting spoilage-associated bacteria, *Pseudomonas* spp. were the most frequently identified microorganisms, reported in 23 studies (76.67%) [12,22,23,24,25,26,27,28,29,30,31,32,33,35,36,38,39,40,41,42,44,45,46]. Total viable count (TVC) was the second most frequently reported microbiological indicator, appearing in 13 studies (43.33%) [12,13,15,21,22,23,26,29,39,41,42,45,47]. Lactic acid bacteria, including *Leuconostoc* spp. and *Lactobacillus* spp., were identified in 10 studies (33.33%) [12,13,23,29,37,39,41,42,45,47], while spore-forming bacteria (*Bacillus* spp. and *Clostridium* spp.) were reported in 2 studies (6.67%) [34,37].

Additional taxa reported included coliforms [12,13,23,28,29,37,42,45,46,47], Enterococci [23,33], *Micrococcaceae*, Streptococci [23], *Staphylococcus* spp. [21,33], and other genera such as *Acinetobacter*, *Aeromonas*, *Burkholderia*, *Citrobacter*, *Enterobacter*, *Escherichia*, *Kluyvera*, *Ochrobactrum*, *Pasteurella and Proteus* [43].

Table 4 summarizes the main spoilage-associated bacterial species in various types of fresh cheeses, highlighting the diversity of microorganisms across cheese varieties.

Across the included studies, Gram-negative psychrotrophic bacteria, particularly *Pseudomonas* spp. and members of the *Enterobacteriaceae*, were the dominant spoilage microbiota. In *Fior di latte*, *P. fluorescens* was identified as a key spoilage species. Soft, white, fresh/fresco cheeses exhibited high diversity, including species such as *P. pseudoalcaligenes*, *P. alcaligenes*, *P. aeruginosa*, *P. fragi*, *P. fluorescens*, *P. shahriarae*, *P. koreensis*, *P. veronii*, *P. paracarnis*, *P. lundensis*, and *P. solani*. In Mozzarella, species such as *P. fragi*, *P. lundensis*, *P. taetrolens*, *P. gessardii*, and *P. fluorescens* were identified, while *P. fluorescens* was reported in Fior di latte cheese.

*Enterobacteriaceae* were detected across a wide range of fresh cheese varieties, particularly Mozzarella. The detected taxa included *Rahnella aquatilis*, *Enterobacter amnigenus*, *Hafnia alvei*, *Buttiauxella* spp., *Cedecea davisae*, *Citrobacter freundii*, *Kluyvera* spp., *Serratia* spp., *Pantoea* spp., *Raoultella* spp., *Klebsiella pneumoniae*, and *Escherichia coli*. Minas cheese contained *Citrobacter freundii*, *Enterobacter* spp., *E. coli*, and *Hafnia alvei*. Similarly, Oaxaca, Panela, and Cottage cheeses exhibited diverse *Enterobacteriaceae*, particularly *Enterobacter*, *Klebsiella*, *Serratia*, and *Citrobacter* species.

Beyond Gram-positive spoilage bacteria, lactic acid bacteria also contributed to the spoilage microbiota of several fresh cheese varieties and were frequently detected during refrigerated storage. Reported taxa included *Enterococcus faecalis*, *Enterococcus pseudoavium*/*devriesei*, *Lactobacillus graminis*, *Lacticaseibacillus paracasei*, *Lactiplantibacillus paraplantarum*, and several *Leuconostoc species*, such as *L. mesenteroides* and *L. dextranicum*. Additional species included *Carnobacterium gallinarum* and *Lactobacillus fermentum*. In Oaxaca, Panela, and Cottage cheeses, genera such as *Lactobacillus*, *Streptococcus*, *Lactococcus*, and *Leuconostoc* were identified, including *Streptococcus thermophilus* and *Lactococcus lactis* subspecies.

Although less commonly reported, spore-forming bacteria were associated with specific spoilage events and technological defects in soft cheeses, often linked to gas production and structural deterioration. Species such as *Paenibacillus polymyxa* (reported as *Bacillus polymyxa*), *Paenibacillus macerans* (reported as *Bacillus macerans*), and *Clostridium tyrobutyricum* were reported.

### 3.3. Physicochemical and Reported Sensory Alterations Associated with Bacterial Spoilage

Physicochemical changes included a reduction in pH during storage, reported in seven studies (23.33%) [13,28,29,33,39,42,47]. Sensory alterations associated with fresh cheese spoilage were heterogeneous, affecting multiple quality attributes, including odor, flavor, texture, color, appearance, and overall acceptability, across 10 studies (33.33%) [22,23,33,38,39,40,41,42,46,47]. Discoloration was also identified as a recurrent spoilage-related defect in six studies (20%) [15,30,32,35,36,37].

Microbial activity was associated with progressive physicochemical and biochemical changes during storage. In particular, pH decreased over time, declining from 5.8 to 6.0 in the initial stages to 4.8–5.2 by the end of storage in Stracciatella cheese [42]. In Domokou-type cheese, both moisture content and water activity (a_w_) increased, from 72% to 78% and from 0.88 to 0.92, respectively.

Biochemical changes associated with microbial metabolism were also observed. One study reported lactose depletion accompanied by the production of lactic and acetic acids [46], while proteolytic and lipolytic activity was reported in four studies [21,26,31,44]. These processes may contribute to nutrient degradation, compromise the structural integrity of the cheese matrix, and promote the development of off-odors and off-flavors.

A decline in sensory quality was characterized by reduced odor and flavor intensity, sour or atypical odor and taste notes, loss of consistency, surface deterioration, and decreased overall acceptability (33.33%, n = 10) [22,23,33,38,39,40,41,42,46,47]. Visual spoilage defects were also prominent, including surface smear, gas formation, package blowing, and gas-induced openings [33].

Visual defects represented another important category of spoilage-related changes. Discoloration phenomena included dark blue, gray, yellow, creamy, orange-brown, blue, slightly blue, and gold or yellow-brown discoloration (n = 5, 16.67%) [15,30,32,35,36]. Overall, sensory deterioration was consistently associated with microbial growth and emerged as a key factor contributing to product rejection and reduced overall quality.

### 3.4. Quantification and Identification Methods for Spoilage Bacteria

Various methodological approaches were used to quantify and identify spoilage-associated bacteria in fresh cheeses, broadly classified into culture-based and molecular methods. Culture-based techniques were primarily used for microbial enumeration and preliminary identification, whereas molecular methods provided higher taxonomic resolution and confirmed bacterial identity. Several studies combined both approaches to integrate quantitative analysis with molecular characterization.

Culture-dependent methodologies were the primary approach for quantifying and isolating pure cultures, enabling the preliminary identification of spoilage microbiota in fresh cheeses, as reported in 28 of 30 studies (93.33%) (e.g., refs. [12,21,25,31,47]).

Spoilage bacterial counts were quantified using both non-selective and selective media (Table 5). Total viable counts were determined using Plate Count Agar (PCA), whereas selective enumeration targeted specific microbial groups, including *Pseudomonas* spp. using Pseudomonas agar, and *Enterobacteriaceae* using Violet Red Bile Glucose Agar (VRBG). Additional media included Violet Red Bile Lactose or Petrifilm Plate for coliform bacteria, and de Man, Rogosa, and Sharpe (MRS) Agar for lactic acid bacteria.

Biochemical characterization was applied in five studies (16.67%), including morphological assessment, Gram staining, motility, oxidase production, gelatin hydrolysis, tryptophan hydrolysis (indole production), utilization of carbohydrates (xylose, trehalose, glucose, arabinose, mannitol, galactose, fructose, and sucrose), and nitrate reduction [31]. Within this category, the Bactray I, II, and III Kit, as well as the API 20E and API 20NE systems, were also used [12,15,43].

Molecular approaches were reported in 6 studies (20%), primarily based on 16S rRNA gene sequencing and PCR assays for taxonomic confirmation and species-level identification. Advanced molecular typing techniques, including RAPD-PCR, PFGE, and MLST, were employed in 3 studies (10%) to assess strain-level diversity and contamination sources.

High-throughput sequencing approaches, such as 16S amplicon sequencing and whole-genome sequencing (WGS), were applied in 3 studies (10%), enabling the detection of non-culturable taxa and comprehensive microbial profiling. Combined methodological strategies integrating culture-dependent and molecular approaches were observed in 10 studies (33.33%), enabling simultaneous quantification and high-resolution identification. 

Microbial loads at the end of shelf life in untreated cheeses showed wide variability across cheese matrices, with total viable counts ranging from 6.37 to 9.33 log CFU/g. The highest levels were observed in Mozzarella (7.70–9.33 log CFU/g), whereas lower counts were reported in Fresh cheese (6.5–8 log CFU/g). Intermediate values were identified in Mexican varieties, including Oaxaca, Panela, and Cottage cheeses (6.37–8.46 log CFU/g). A consistent trend was the predominance of lactic acid bacteria during the advanced stages of storage, with counts reaching 8.25–8.31 log CFU/g in Oaxaca/Panela/Cottage cheese, indicating their prevalence during late spoilage (Table 6).

Among spoilage-associated microorganisms, *Pseudomonas* spp. and *Enterobacteriaceae* exhibited broad, overlapping ranges. *Pseudomonas* spp. counts ranged from 2.78 to 8.04 log CFU/g, with higher levels observed in Fior di latte and Stracciatella cheeses (up to 6 log CFU/g).

With respect to *Enterobacteriaceae*, counts ranged from 2.9 to 8.04 log CFU/g, reflecting substantial variability among cheese types, with the highest levels detected in Mozzarella and Stracciatella cheeses. Coliform bacteria were consistently detected at levels between 3 and 7 log CFU/g, whereas spore-forming bacteria, such as *Clostridium* and *Bacillus* spp., were generally present at lower concentrations (5–6 log CFU/g) and were associated with specific defects, such as blowing. Overall, the available evidence suggests a progressive increase in microbial loads during storage, with Gram-negative bacteria contributing primarily to the early and intermediate stages of spoilage, whereas lactic acid bacteria predominated in the later stages.

## 4. Discussion

The present review synthesized evidence on the diversity of spoilage-associated bacterial communities in fresh cow cheeses, their associations with physicochemical changes, reported sensory alterations, and shelf-life reduction, and the methodologies used for their identification and quantification. Beyond confirming established roles of major spoilage-associated bacterial groups, this review provides a structured mapping of how these bacteria have been investigated across fresh cow cheese types, the spoilage-related changes reported in association with them, and the distribution of culture-dependent, molecular, and high-throughput approaches. The evidence also reveals substantial methodological heterogeneity and limited integration of advanced sequencing approaches and formal sensory assessments, which constrain cross-study comparability and a comprehensive characterization of spoilage dynamics.

A consistent pattern was observed; psychrotrophic Gram-negative bacteria, particularly *Pseudomonas* spp. and *Enterobacteriaceae*, were the predominant spoilage-associated microorganisms in high-moisture fresh cheeses. These microorganisms were consistently detected in Mozzarella, Fior di latte, Minas, Stracciatella, Burrata, and other fresh or white cheese varieties.

The total viable count (TVC) frequently reached high microbial loads, supporting its value as a general indicator of microbial proliferation during fresh cheese spoilage. Additional microbiological quality and hygiene indicators, such as *Escherichia coli* and *Staphylococcus aureus*, are commonly quantified because of their relevance to fresh cheese spoilage. High counts of these indicators may reflect contamination originating from the processing environment, raw materials, food handlers, or other factors associated with the manufacturing process. Overall, high microbial loads are generally associated with evident spoilage, as these microorganisms metabolize nutrients available within the cheese matrix, resulting in physicochemical and sensory deterioration [48].

Culture-dependent methods were the primary approach used to study spoilage microbiota in fresh cheeses, reported in 28 of 30 studies, primarily for microbial enumeration (CFU/g or Log CFU/g) and preliminary identification. The use of molecular techniques was comparatively limited, primarily for taxonomic confirmation via PCR and 16S rRNA sequencing, whereas advanced typing methods and high-throughput approaches, such as amplicon sequencing and WGS, were used in a limited number of studies.

Overall, the reviewed studies demonstrated that microbial spoilage was associated with physicochemical and sensory changes, including pH reduction, proteolysis, lipolysis, discoloration, texture deterioration, gas formation, lactic and acetic acid production, off-flavors, and unpleasant odors, ultimately leading to reduced shelf life and product rejection.

Psychrotrophic *Pseudomonas* species can grow at refrigeration temperatures and produce extracellular proteases, slime formation, lipases, and pigments that negatively affect dairy product quality [15,49,50,51]. The structural stability and significant residual activity of these extracellular enzymes under refrigerated storage conditions may contribute to the continuous degradation of cheese components and, consequently, to food spoilage [52,53].

The recurrent detection of *P. fluorescens* in the included studies supports its role as a key spoilage-associated species in these cheese varieties [15,27,32,38]. Further support for this interpretation comes from studies identifying *P. fluorescens* as the causative agent of blue discoloration defects in fresh cheeses and broader evidence linking the *P. fluorescens* group to dark blue, gray, yellow, creamy, orange-brown, blue, slightly blue, and gold or yellow-brown pigmentation defects in these foods [15,49]. Therefore, the frequent occurrence of *Pseudomonas* spp. in Mozzarella, Fior di latte, and related high-moisture cheeses may be explained by their aerobic metabolism, psychrotrophic capacity, enzymatic activity, and potential contamination during post-processing operations such as slicing, brining, packaging, and refrigerated storage [21,24,26,30,40,50,52].

*Enterobacteriaceae* were among the most prevalent groups of spoilage-associated bacteria and were detected across a wide range of fresh cheese varieties, particularly in Mozzarella, Minas, Oaxaca, Panela, and Cottage cheeses. Their occurrence may reflect contamination associated with raw material quality, processing environments, handling, brining, or post-processing operations [12,30,36]. The diversity of genera identified in the included studies, including *Enterobacter*, *Klebsiella*, *Serratia*, *Rahnella*, *Hafnia*, *Citrobacter*, and *Pantoea*, highlights the microbiological complexity of fresh cheese ecosystems [12,26,43].

These *Enterobacteriaceae* may contribute to spoilage through gas production, proteolytic activity, formation of volatile compounds, and sensory deterioration, although their specific contributions vary with the cheese matrix and storage conditions [38,43,52]. Variability among studies regarding microbial counts and taxonomic composition may be explained by differences in cheese composition, hygienic practices, heat treatment, storage temperature, packaging system, and microbiological methods used for enumeration and identification [12,22,24,28,38,39,40,41,42].

The predominance of LAB during late spoilage may contribute to sour or fermented odors and reduced sensory acceptability [33,37]. Similarly, the occurrence of *Clostridium* spp. and *Bacillus* spp. in some cheeses was associated with gas formation and blowing defects [34,37]. These defects are technologically relevant because spore-forming bacteria may survive adverse conditions and proliferate during storage when the cheese matrix provides favorable conditions [34].

Although the included studies did not explicitly assess microbial succession through longitudinal monitoring of community composition, temporal growth patterns and associated microbiological and sensory changes suggest potential ecological trajectories during the spoilage of certain fresh cheeses, particularly Mozzarella and Stracciatella.

In refrigerated Mozzarella, available findings suggest an initial expansion of the total bacterial microbiota, followed by the proliferation of spoilage-associated psychrotrophs, particularly *Pseudomonas* spp., which frequently reach levels that limit shelf life. Coliforms and other members of *Enterobacteriaceae* also increase, whereas lactic acid bacteria (LAB) and enterococci may persist or increase concurrently. Thus, the observed patterns do not support a simple linear replacement of Gram-negative bacteria by LAB but rather suggest overlapping growth trajectories and shifts in relative dominance shaped by storage temperature and preservation strategies [22,23].

In Stracciatella, the findings likewise indicate differential growth dynamics rather than demonstrated microbial succession. These patterns are characterized by the initial predominance of *Pseudomonas* spp., with subsequent increases in coliforms, *Enterobacteriaceae*, and other psychrotrophs, whereas LAB remain relatively stable and may contribute to the late-stage decline in pH [42]. In Minas Frescal cheese, the coexistence of *Acinetobacter*, *Burkholderia*, *Hafnia*, *Pseudomonas*, *Enterobacter*, *Serratia*, and other genera could reflect taxonomic diversity and the concurrent presence of psychrotrophic bacteria rather than microbial succession [43].

Oxygen availability may also influence community structure, particularly because aerobic psychrotrophs such as *Pseudomonas* spp. can be favored on oxygen-exposed surfaces, whereas reduced oxygen availability within the cheese matrix or under packaging may alter microbial competition [44]. Therefore, the observed growth patterns likely reflect interacting effects of storage temperature, acidification, substrate availability, oxygen conditions, and microbial competition.

However, none of the included studies specifically characterized microbial succession in these products through longitudinal community-level analyses. This gap limits a comprehensive understanding of how microbial populations emerge, coexist, shift in relative dominance, or are replaced throughout storage and spoilage. It represents an important limitation of the current literature and highlights the need for longitudinal studies specifically designed to characterize microbial succession patterns and elucidate the ecological dynamics underlying community changes during fresh cheese spoilage.

Differences in these intrinsic and extrinsic factors among fresh cheese types may therefore create distinct selective environments, contributing to variation in spoilage-associated microbial populations across cheeses. Additionally, raw material quality, milk composition and blending, and cheese-specific manufacturing processes may influence the structure and dynamics of spoilage-associated microbial communities [2,3,16].

The physicochemical and sensory changes identified in the included studies are consistent with the metabolic activity of spoilage microbiota. Proteolytic and lipolytic processes led to nutrient degradation, weakening of the cheese matrix, and the generation of volatile compounds that contribute to off-flavors and unpleasant odors [22,23,38,39,41]. The reduction in pH observed during storage likely reflects lactose metabolism and the production of organic acids by LAB and other fermentative microorganisms [39,46].

In addition, discoloration phenomena, changes in texture, softening, loss of elasticity, and gas production may result from pigment synthesis, exopolysaccharide production, enzymatic degradation, and fermentative metabolism [15,27,33,37,49]. These alterations are not only microbiological outcomes but also commercially relevant quality defects because they directly affect consumer perception, shelf life, product rejection, and food waste [15,22,27,28,38,51].

Regarding methodological approaches, culture-dependent methods remained the predominant tools for microbial quantification and preliminary identification. These approaches remain useful because they provide viable counts, allow isolation of pure cultures, and enable phenotypic or biochemical characterization of spoilage traits, such as proteolytic and lipolytic activities.

Molecular methods were less frequently reported despite their importance; these included PCR, 16S rRNA gene sequencing, RAPD-PCR, PFGE, MLST, next-generation sequencing, and whole-genome sequencing, which can improve taxonomic resolution and the identification of specific spoilage taxa or contamination sources [12,15,21,30,32,33].

This methodological distribution should be interpreted in the context of the 1992–2025 period covered by the included evidence. Culture-dependent approaches were reported throughout this period, whereas molecular and high-throughput sequencing methods were mainly reported in more recent studies, consistent with the progressive incorporation of these tools into food microbiology research [54]. However, because no formal stratified analysis by publication period or geographical region was performed, the influence of these factors on methodological choice cannot be established.

The integration of culture-dependent and molecular methods, as observed in several studies, is therefore a methodological strength because it enables complementary assessment of viable counts, strain isolation, taxonomic confirmation, and source tracking [2].

Each methodological approach nevertheless provides distinct information on spoilage ecosystems. Culture-dependent methods remain essential for viable enumeration, strain isolation, phenotypic characterization, and experimental confirmation of spoilage traits [55], but microbial recovery is influenced by medium composition, incubation temperature, atmosphere, and the cells’ physiological state.

Targeted molecular methods improve taxonomic confirmation and source tracking but remain restricted to predefined targets and may overlook unexpected community members. High-throughput sequencing provides broader community-level characterization and can reveal taxa not recovered under the applied culture conditions [56]; however, its interpretation may be affected by DNA extraction and amplification biases, limited discrimination between viable and non-viable cells, and, depending on the sequencing strategy, insufficient species- or strain-level resolution [57]. Consequently, no single approach provides a complete representation of spoilage microbiota, supporting the complementary use of culture-dependent, targeted molecular, and sequencing-based methods [18,58].

Although reported in four studies, next-generation sequencing enabled broader characterization of spoilage microbiota and revealed shifts in microbial community structure during storage. Beyond taxonomic profiling, these approaches may help investigate microbial succession and contamination patterns across the cheese production chain [59]. Consequently, next-generation sequencing has become an increasingly valuable tool for characterizing food microbiota and spoilage ecosystems [54,60,61,62].

This scoping review has several strengths. It compiled evidence from multiple fresh cheese varieties produced across different geographical regions, providing a broad overview of spoilage microbiota in high-moisture cheeses. It integrated microbiological, physicochemical, sensory, shelf-life, and methodological evidence, allowing a multidimensional interpretation of spoilage in fresh cheeses.

Several limitations should be considered when interpreting the findings of this review. Considerable heterogeneity existed among studies with respect to cheese type, packaging conditions, storage temperature, microbial targets, culture media, molecular methods, and spoilage indicators. In addition, many studies relied primarily on culture-dependent analyses, whereas relatively few employed high-throughput sequencing approaches to characterize taxa not recovered under the applied culture conditions, to characterize microbial community interactions, or to resolve strain-level diversity using whole-genome sequencing [24,44,47]. Because culture-based, targeted molecular, and high-throughput sequencing methods differ in breadth of detection and taxonomic resolution, they may partly reflect the most frequently applied methodological approaches rather than a complete representation of the spoilage microbiota.

This is particularly relevant given the absence of a formal critical appraisal or risk-of-bias assessment, which precludes determining whether individual findings are based on comparatively stronger or weaker study designs. Accordingly, the synthesized patterns should be interpreted as a mapping of the available evidence rather than as a hierarchy of microbial prevalence or methodological accuracy.

Despite these limitations, the findings of this scoping review could have valuable implications for food microbiology, dairy technology, quality control, and shelf-life management. The predominance of psychrotrophic Gram-negative bacteria underscores the importance of improving hygiene practices during milk handling, cheese manufacturing, brining, slicing, packaging, and refrigerated storage [21,24,26,30,50,52].

Identifying specific deterioration taxa and their associated defects can support research on targeted conservation strategies, including modified-atmosphere packaging, active coatings, protective cultures, natural antimicrobials, and integrated-hurdle approaches. In addition, the increasing application of molecular and high-throughput sequencing techniques may improve monitoring of spoilage microbiota and contamination sources in dairy production chains [2,24,44,47].

Future studies should prioritize standardized experimental designs, longitudinal microbiome analyses, strain-level tracking, predictive microbiology, and integrative approaches that combine metagenomics, metabolomics, enzymatic assays, and sensory analysis to better understand microbial succession and spoilage mechanisms in fresh cheeses.

## 5. Conclusions

This scoping review mapped the available evidence on spoilage-associated bacteria in fresh cow cheeses and showed that psychrotrophic Gram-negative bacteria, particularly *Pseudomonas* spp. and members of the *Enterobacteriaceae*, were the predominant bacterial groups reported across the included studies. Lactic acid bacteria and spore-forming bacteria, including *Bacillus* spp. and *Clostridium* spp., were also associated with spoilage phenomena. Collectively, these bacterial groups were linked to physicochemical and sensory alterations, including proteolysis, lipolysis, acidification, slime formation, discoloration, gas production, texture deterioration, off-flavors, and unpleasant odors, ultimately contributing to reduced shelf life and product rejection.

Spoilage patterns varied across cheese types and storage conditions. Mozzarella, Fior di latte, and Stracciatella were the cheese types most frequently associated with psychrotrophic Gram-negative bacteria in the included evidence, highlighting the relevance of post-processing contamination and refrigerated storage in spoilage development. These findings support the importance of hygienic control and Good Manufacturing Practices (GMP) throughout milk handling, cheese processing, brining, slicing, packaging, and cold storage to limit contamination and delay quality deterioration.

Culture-dependent methods remained the predominant approaches for bacterial enumeration, isolation, and preliminary identification, whereas molecular and high-throughput sequencing methods were less frequently reported but provided higher taxonomic resolution and improved characterization of bacterial diversity and potential contamination sources. The combined use of culture-dependent and molecular approaches may therefore provide a more comprehensive assessment of spoilage-associated bacteria. Future studies should prioritize standardized longitudinal designs, strain-level tracking, and integrated molecular, metagenomic, metabolomic, enzymatic, and formal sensory approaches to better characterize bacterial succession, spoilage mechanisms, and their relationships with quality deterioration in fresh cheeses.

## Figures and Tables

**Table 1:** Search strategies applied across the selected databases.

Database	Search Strategy
PubMed	(“Cheese”[MeSH Terms] OR “Dairy Products”[MeSH Terms] OR cheese[Title/Abstract] OR “fresh cheese”[Title/Abstract] OR “soft cheese”[Title/Abstract] OR “unripened cheese”[Title/Abstract]) AND (“Food Microbiology”[MeSH Terms] OR spoilage[Title/Abstract] OR deteriorat*[Title/Abstract] OR “microbial spoilage”[Title/Abstract] OR deterioration[Title/Abstract]) AND (“Bacteria”[MeSH Terms] OR “Microbiota”[MeSH Terms] OR bacter*[Title/Abstract] OR microbi*[Title/Abstract] OR microbiota[Title/Abstract] OR microflora[Title/Abstract] OR bacteria[Title/Abstract])
Scopus	TITLE-ABS-KEY((cheese OR “fresh cheese” OR “soft cheese” OR “unripened cheese”) AND (spoilage OR deteriorat* OR “food spoilage” OR “microbial spoilage” OR deterioration) AND (bacter* OR microbi* OR microbiota OR microflora OR bacteria))
Scielo	(“queso fresco” OR cheese OR “fresh cheese” OR “queso blando”) AND (deterioro OR spoilage OR deteriorat* OR “deterioro microbiano”) AND (bacterias OR bacteria OR microbiologia OR microbiología OR microbiology OR microbiota)
Google Scholar *	Equation 1: “fresh cheese” spoilage bacteria microbiology Equation 2: (“fresh cheese” OR “queso fresco”) spoilage bacteria deterioro microbiology microbiología Equation 3: cheese spoilage bacteria microbiology “fresh cheese” “queso fresco”

* A predefined stopping rule specified in the registered review protocol was applied: the first 100 records retrieved for each Google Scholar search equation were screened; when fewer than 100 records were retrieved, all available records were screened.

**Table 2:** The inclusion and exclusion criteria for the articles selected.

Inclusion Criteria	Exclusion Criteria
– Cow cheese type: Fresh, high-moisture, unripened, and short shelf-life cheeses. – Microorganisms: Spoilage-associated bacteria, including those responsible for defects and shelf-life reduction. – Methodologies: Classical and molecular techniques for identification and quantification, other. – Outcomes: Bacterial identification and counts, physicochemical and sensory changes, shelf-life reduction, technological defects, and organoleptic alterations. – Study types: Experimental, microbiological, observational, shelf-life, quality, spoilage, storage, bacterial identification, and sustainability-related studies. – Document types: Peer-reviewed original research articles indexed in regional and high-impact journals.	– Document type: Books, book chapters, theses, dissertations, preprints, and review articles. – Full-text unavailable: Studies lacking full-text access or sufficient data. – Microorganisms type: Yeasts, molds, probiotics, starter cultures, beneficial microorganisms, and pathogens not associated with spoilage. – Bacterial identification: Studies not reporting bacterial identification or quantification, or lacking descriptions of bacterial spoilage. – Scope: Studies unrelated to spoilage, including those focused on controlled fermentation, product development, technological improvements, or food safety without spoilage relevance.

**Table 3:** Summary of the characteristics of the included studies.

No.	Study Title	Cheese Type	Spoilage-Associated Bacteria or Bacterial Groups Reported	Main Methodological Approach	Main Spoilage-Related Finding	Reference
1	The main spoilage-related psychrotrophic bacteria included in the industrial slicing of mozzarella cheese under sanitation standard operating procedures.	Mozzarella.	*Staphylococcus* spp.	Psychrotrophic enumeration, spoilage potential assays, genetic diversity analysis, partial 16S rRNA gene sequencing.	No significant change in psychrotrophic counts after slicing, but introduction of low-level environmental and handling-related bacteria with spoilage potential.	[21]
2	Shelf life extension of Italian mozzarella by use of calcium lactate buffered brine.	Mozzarella.	Total mesophilic bacteria, *Pseudomonas* spp. and *Enterobacteriaceae*.	Acidified calcium lactate brine intervention, refrigerated shelf-life study and microbial counts and physicochemical and sensory analyses.	Delayed growth of spoilage bacteria, prevention of blue discoloration, and extension of shelf life to 21 days.	[22]
3	Antimicrobial efficacy of a polyphenolic extract from olive oil by-product against Fior di latte cheese spoilage bacteria.	Fior di latte.	*Enterobacteriaceae* and *Pseudomonas fluorescens*.	Polyphenolic extract intervention during refrigerated storage, microbial growth modeling and sensory shelf-life analysis.	Delayed growth of *P. fluorescens* and *Enterobacteriaceae*.	[38]
4	Use of chitosan to prolong mozzarella cheese shelf life.	Mozzarella.	Coliforms, *Pseudomonas* spp., Enterococci, *Micrococcaceae*, mesophilic and thermophilic lactic acid bacilli and lactic acid streptococci.	Chitosan intervention during refrigerated storage, microbial enumeration and predictive shelf-life modeling and sensory evaluation	Inhibited coliform and *Pseudomonas* spp. growth, increased predicted shelf life, and better maintained texture.	[23]
5	Spoilage potentials and antimicrobial resistance of *Pseudomonas* spp. isolated from cheeses.	Cheese (fresh/white cheese).	*Pseudomonas* spp.	Selective isolation, biochemical identification and extracellular spoilage-enzyme assays.	Widespread protease and lecithinase production and frequent lipase activity among cheese-derived *Pseudomonas* isolates.	[31]
6	Shelf life of Stracciatella cheese under modified-atmosphere packaging.	Stracciatella.	Total viable count, *Enterobacteriaceae*, total coliforms, LAB, and *Pseudomonas* spp.	MAP storage trial, microbial counts and sensory evaluation.	MAP delayed spoilage and bacterial growth, with M1 and M2 prolonging sensory acceptability.	[42]
7	The microbiota of high-moisture mozzarella cheese produced with different acidification methods.	Mozzarella.	Psychrotrophic spoilage microorganisms.	16S rRNA gene pyrosequencing and microbial community analysis.	Psychrotrophic spoilage taxa were widespread, with higher abundance in directly acidified cheeses and substantial lot-to-lot variability.	[24]
8	The effect of incorporating calcium lactate in the saline solution on improving the shelf life of Fiordilatte cheese.	Fior di latte.	*Pseudomonas* spp., *Enterobacteriaceae* and LAB (Lactic Acid Bacteria).	Culture-dependent microbial enumeration and quantitative descriptive sensory analysis.	*Pseudomonas* spp. increased during refrigerated storage and reached spoilage-relevant levels; calcium lactate showed only a modest, transient inhibitory effect, while odor deterioration was the main shelf-life-limiting sensory change.	[39]
9	Surface UV-C light treatments to prolong the shelf-life of Fiordilatte cheese.	Fior di latte.	*Pseudomonas* spp. and *Enterobacteriaceae*.	UV-C surface treatment, refrigerated shelf-life study, microbial enumeration and sensory evaluation.	UV-C treatment reduced initial *Pseudomonas* spp. and *Enterobacteriaceae* loads and extended shelf life, with the highest tested effective treatment achieving an 80% extension.	[40]
10	Study on the combined effects of essential oils on microbiological quality of Fior di Latte cheese.	Fior di latte.	Total count bacteria, LAB, *Pseudomonas* spp and coliforms.	Essential-oil intervention using a central composite design, refrigerated storage and microbial enumeration.	Essential-oil combinations delayed the growth of *Pseudomonas* spp. and coliforms and increased microbiological stability.	[45]
11	Effect of modified atmosphere packaging on the growth of spoilage microorganisms and *Listeria monocytogenes* on fresh cheese.	Fresh cheese.	Total count bacteria, Coliform, and Lactic Acid Bacteria count.	Modified-atmosphere packaging intervention, refrigerated storage and microbial enumeration	Increasing CO_2_ concentration progressively inhibited spoilage microbial growth, with 70–100% CO_2_ showing the strongest control during storage	[13]
12	Diversity and spoilage potential of *Pseudomonas* spp. from Spanish milk and dairy products: Impact on fresh cheese and milk quality.	Fresh cheese.	*Pseudomonas* spp.	Isolation and polyphasic strain characterization, molecular identification, in vitro spoilage assays, and simulated fresh cheese.	*Pseudomonas* spp. showed marked strain-dependent spoilage potential, causing diverse discolorations in fresh cheese and substantial proteolysis under refrigerated conditions.	[32]
13	*Klebsiella pneumoniae* as a spoilage organism in mozzarella cheese	Mozzarella.	*Klebsiella pneumoniae*, total and fecal coliform counts.	Microbiological enumeration, phenotypic identification and inoculation challenge test with gas quantification	*Klebsiella pneumoniae* was identified as the predominant spoilage organism and experimentally linked to gas production, package swelling, and gas-hole formation.	[25]
14	Packaging optimisation to prolong the shelf life of fiordilatte cheese	Fior di latte.	*Pseudomonas* spp. *Enterobacteriacea*, LAB and total count bacteria.	Sequential MAP screening, edible/active coating trials during refrigerated storage and microbiological and sensory monitoring.	High-CO_2_ MAP controlled *Pseudomonas* spp. growth but impaired texture, whereas coating protected cheese quality and, when combined with active coating and MAP, extended shelf life by about 157%.	[41]
15	Occurrence of non-lactic acid bacteria populations involved in protein hydrolysis of cold-stored high moisture Mozzarella cheese	Mozzarella.	Total microbial count, *Pseudomonas*, *Acinetobacter*, *Rahnella*.	Culture-dependent enumeration and isolation, molecular biotyping and identification, proteolytic activity assays and inoculated cold-storage challenge test.	Proteolytic non-LAB strains caused strain-dependent casein hydrolysis, mainly at the cheese surface, associated with visible wrinkling, exfoliation, cheese debris, and increased turbidity.	[26]
16	Lactic Acid Bacteria Adjunct Cultures Exert a Mitigation Effect against Spoilage Microbiota in Fresh Cheese	Fresh cheese.	Staphylococci, Enterococci, heterofermentative lactobacilli, *Pseudomonas*, *Enterobacteriaceae* and *Streptococcus thermophilus*.	Culture-dependent microbiological analysis and strain isolation, molecular fingerprinting and 16S rRNA identification, in vitro inhibition assays and industrial challenge trials.	Psychrotrophic Gram-negative bacteria dominated the spoilage microbiota and were associated with surface smear, gas defects, discoloration, off-flavors, and package blowing; selected LAB adjunct cultures reduced spoilage populations.	[33]
17	*Pseudomonas fluorescens* and *Escherichia coli* in Fresh Mozzarella Cheese: Effect of Cellobiose Oxidase.	Mozzarella.	*Pseudomonas fluorescens*.	Microbial challenge tests during refrigerated storage, microbial enumeration and H_2_O_2_ quantification.	Cellobiose oxidase strongly inhibited *E. coli* throughout storage but only partially and transiently affected *P. fluorescens*.	[27]
18	Application of Commercial Biopreservation Starter in Combination with MAP for Shelf-Life Extension of Burrata Cheese.	Burrata.	*Pseudomonas*, *Enterobacteriaceae* and total coliforms.	Biopreservation starter and modified-atmosphere packaging intervention, refrigerated shelf-life challenge study, and microbiological, chemical, and sensory analyses.	Combined bioprotective starter and MAP reduced spoilage-associated bacterial counts and delayed lactose, citric acid, lipolytic, and VOC changes, preserving sensory quality and extending Burrata shelf life by about 7 days.	[46]
19	Use of active compounds for prolonging the shelf life of mozzarella cheese.	Mozzarella.	Coliforms, *Pseudomonadaceae* and LAB.	Lysozyme–Na_2_-EDTA intervention in conditioning brine, refrigerated storage, microbial enumeration, and predictive shelf-life modeling.	Lysozyme–Na_2_-EDTA inhibited coliforms and *Pseudomonas* spp. without affecting LAB, extending microbiologically determined shelf life.	[28]
20	Application of Natural Antimicrobial Additives and Protective Culture to Control Aerobic Spore Forming Bacteria in Low Salt Soft Cheese.	Soft cheese.	Aerobic spore-forming bacteria (*Bacillus* spp.).	Natural antimicrobial intervention, refrigerated storage and enumeration of aerobic spore-forming bacteria and in vitro inhibition assay.	Nisin–lysozyme treatment produced the greatest reduction in aerobic spore-forming bacteria during refrigerated storage.	[34]
21	Effect of Immobilized *Pediococcus acidilactici* ORE5 Cells on Pistachio Nuts on the Functional Regulation of the Novel Katiki Domokou-Type Cheese Microbiome.	Katiki Domokou-type.	*Enterobacteriaceae*, coliforms, LAB, and total viable count.	Probiotic biopreservation intervention, refrigerated storage, culture-dependent microbiology, 16S rRNA NGS microbiome profiling, HS-SPME GC/MS and sensory evaluation.	*P. acidilactici* reduced spoilage-associated genera and improved the volatile and sensory profile of cheeses.	[47]
22	A food-grade resin with ldh–salicylate to extend mozzarella cheese shelf life.	Mozzarella.	Total count bacteria, *Pseudomonas* spp. and LAB.	Active antimicrobial packaging intervention, refrigerated and thermal-abuse storage, culture-dependent microbiological enumeration, and physicochemical analyses.	Salicylate–LDH active packaging reduced spoilage-associated microbial growth, particularly *Pseudomonas* spp. and coliforms, and slowed acidification and proteolysis.	[29]
23	Psychrotrophic bacteria in Brazilian organic dairy products: identification, production, and spoilage potential.	Minas.	*Acinetobacter*, *Aeromonas*, *Burkholderia*, *Citrobacter*, *Enterobacter*, *Escherichia*, *Kluyvera*, *Ochrobactrum*, *Pasteurella*, *Proteus*, *Pseudomonas* spp.	Culture-dependent enumeration of psychrotrophic microorganisms, phenotypic/biochemical identification, 16S rRNA gene sequencing, extracellular spoilage-enzyme assays.	Minas Frescal cheese showed high psychrotrophic counts; Gram-negative psychrotrophs, while cheese isolates exhibited spoilage-related protease, lecithinase, and lipase production potential.	[43]
24	Cinnamon Essential Oil and Nanoemulsions for Inhibiting *Pseudomonas paracarnis* and Pigment Production in Fresh Cheese.	Fresh cheese.	*Pseudomonas* spp.	Selective culture-based enumeration of inoculated *Pseudomonas paracarnis*, photographic monitoring of pigment development and antimicrobial intervention with cinnamaldehyde, free cinnamon essential oil, and nanoemulsified essential oil.	*Pseudomonas paracarnis* caused progressive gray-to-blue discoloration in untreated model fresh cheese during refrigerated storage; the untreated control developed a grayish hue from day 4, accompanied by marked instrumental color changes.	[35]
25	*Pseudomonas* spp. and other psychrotrophic microorganisms in inspected and non-inspected Brazilian Minas Frescal cheese: proteolytic, lipolytic and AprX production potential.	Minas.	*Pseudomonas* spp.	Most psychrotrophic and *Pseudomonas* isolates showed proteolytic and/or lipolytic spoilage potential, while *aprX* was detected in 93.5% of molecularly confirmed *Pseudomonas* isolates.	Most psychrotrophic and *Pseudomonas* isolates from Minas Frescal cheese exhibited proteolytic and/or lipolytic spoilage potential, with simultaneous proteolysis and lipolysis detected in 59.9% and 68.1% of isolates, respectively.	[44]
26	Reuterin inhibits *Pseudomonas* spp. growth and biofilm formation and extends the shelf life of fresh cheese.	Fresh cheese.	*Pseudomonas* spp.	Reuterin intervention, in vitro growth and biofilm assays, and inoculated fresh-cheese challenge test.	Reuterin inhibited *Pseudomonas* growth and biofilm formation and prevented or delayed pigmentation defects.	[36]
27	Investigation on the presence of blue pigment-producing Pseudomonas strains along a production line of fresh mozzarella cheese.	Mozzarella.	Blue-pigmenting *Pseudomonas* strains.	Culture-based enumeration, molecular typing, and phylogenetic characterization of Pseudomonas isolates, and experimental confirmation of blue pigment.	Blue-pigmenting *Pseudomonas fluorescens* group strains persisted in processing fluids and caused blue discoloration, indicating a latent risk of contamination along the production line.	[30]
28	Characterization of the microflora of industrial Mexican cheeses produced without added chemical preservatives.	Oaxaca, Panela, Cottage.	Total count bacteria, *Enterobacteriaceae*, *Pseudomonas* spp. and LAB.	Storage study, culture-based enumeration, biochemical and molecular identification of cheese microflora	High levels of LAB and spoilage-associated contaminants were detected after storage, with potential acidification and predominance of *Enterobacteriaceae*.	[12]
29	New and classical spoilage bacteria causing widespread blowing in Argentinean soft and semihard cheeses.	Soft and semihard cheeses.	Lactic acid bacteria, spore-forming bacteria and coliforms.	Survey of blown cheeses, microbial enumeration, isolation, and identification of gas-producing bacteria.	Blowing defects were associated with diverse gas-producing bacteria.	[37]
30	When cheese gets the blues: *Pseudomonas fluorescens* as the causative agent of cheese spoilage	Queso Fresco.	*Pseudomonas fluorescens*.	Microbial enumeration, isolation, molecular identification.	A blue pigment-producing strain of *Pseudomonas fluorescens* caused blue discoloration of fresh cheese.	[15]

**Table 4:** The main bacterial species associated with spoilage in fresh cheeses.

Cheese Type	Bacterial Group or Taxonomic Category	Spoilage-Associated Taxa Reported	Reference
Fior di latte cheese	*Pseudomonas* spp.	*Pseudomonas fluorescens*	[38]
Soft, white, fresh, or fresco cheese.	*Pseudomonas* spp.	*Pseudomonas pseudoalcaligenes*, *P. pseudoalcaligenes subsp. citrulli*, *P. alcaligenes*, *P. aeruginosa*, *P. atacamensis*, *P. fragi*, *P. fluorescens*, *P. shahriarae*, *P. sivasensis*, *P. koreensis*, *P. veronii*, *P. salmasensis*, *P. brennerii*, *P. libanensis*, *P. psychrophila*, *P. gessardii*, *P. proteolytica*, *P. paracarnis*, *P. lundensis*, *P. solani.*	[15,30,31,32,33,35]
Mozzarella cheese	*Pseudomonas* spp.	*Pseudomonas fragi*, *P. lundensis*, *P. taetrolens*, *P.* gessardii, *P. fluorescens.*	[26,27]
Mozzarella cheese	*Enterobacteriaceae*	*Rahnella aquatilis*, *Enterobacter amnigenus*, *Hafnia alvei*, *Buttiauxella agrestis*, *Buttiauxella noackiae*, *Buttiauxella ferragutiae*, *Buttiauxella gaviniae*, *Cedecea davisae*, *Citrobacter freundii*, *Kluyvera cochleae*, *Serratia grimesii*, *Serratia* spp., *Serratia proteamaculans*, *Pantoea* spp., *Raoultella* spp., *Klebsiella pneumoniae*, *Escherichia coli.*	[24,25,26,27]
Minas	*Enterobacteriaceae*	*Citrobacter freundii*, *Enterobacter amnigenus*, *Enterobacter sakazakii* (currently *Cronobacter sakazakii*), *Escherichia coli*, *Hafnia alvei.*	[43]
Oaxaca, Panela, Cottage	*Enterobacteriaceae*	*Enterobacter amnigenus*, *Klebsiella oxytoca*, *Pantoea agglomerans*, *Kluyvera* sp., *Klebsiella pneumoniae*, *Serratia liquefaciens*, *Enterobacter cloacae*, *Enterobacter gergoviae*, *Cronobacter sakazakii* (reported as *Enterobacter sakazakii*), *Serratia marcescens*, *Enterobacter intermedius*, *Citrobacter freundii*, *Klebsiella aerogenes* (reported as *Enterobacter aerogenes*), *Klebsiella ornithinolytica.*	[12]
Fresh, soft cheese	Lactic acid bacteria	*Enterococcus faecalis*, *Enterococcus pseudoavium/devriesei*, *Lactobacillus graminis*, *Lacticaseibacillus paracasei*, *Lactiplantibacillus paraplantarum*, *Leuconostoc pseudomesenteroides*, *Leuconostoc mesenteroides*, *Carnobacterium gallinarum*, *Leuconostoc jonggajibkimchii*, *Leuconostoc dextranicum*, *Leuconostoc mesenteroides* ssp. *dextranicum*, *Lactobacillus fermentum*,	[33,37]
Oaxaca, Panela, Cottage	Lactic acid bacteria	*Lactobacillus* sp., *Streptococcus* sp., *Lactococcus* sp., *Leuconostoc* sp., *Streptococcus thermophilus*, *L. lactis* subsp. *cremoris*, *L. lactis* subsp. *lactis*	[12]
Soft	Spore-forming bacteria	*Paenibacillus polymyxa* (reported as *Bacillus polymyxa)*, *Paenibacillus macerans* (reported as *Bacillus macerans*), *Clostridium tyrobutyricum.*	[37]

Taxa affected by nomenclatural changes are presented using the currently accepted name, with the name reported in the original study indicated in parentheses for traceability.

**Table 5:** Frequency of quantification and identification methods for spoilage-associated bacteria reported across the included studies.

Category	Method	n	%	Reference
Culture-based	Culture-dependent methodologies	28	93.33	[12,13,15,21,22,23,25,26,27,28,29,30,31,32,33,34,35,37,38,39,40,41,42,43,44,45,46,47]
	Total viable counts (Plate Count Agar (PCA))	13	43.33	[12,13,15,21,22,23,26,29,39,41,42,45,47]
	*Pseudomonas* spp. (Pseudomonas Agar (CFC))	23	76.67	[12,22,23,24,25,26,27,28,29,30,31,32,33,35,36,38,39,40,41,42,44,45,46]
	*Enterobacteriaceae* (Violet Red Bilis Agar (VRBA))	9	30	[12,22,23,39,40,41,42,46,47]
	Coliform (Violet Red Bilis Lactose/Petrifilm Plate)	10	33.33	[13,15,23,25,28,37,42,45,46,47]
	LAB (Man Rogosa Sharpe Agar (MRS) or M17 Agar)	10	33.33	[12,13,23,29,37,39,41,42,45,47]
Phenotypic	Biochemical	5	16.67	[12,15,25,31,43]
Molecular	PCR/16S rRNA identification	6	20	[21,26,32,33,43,44]
	Advanced typing (RAPD, PFGE, MLST)	3	10	[15,30,33]
	NGS/WGS	4	13.33	[12,15,24,47]
Combined	Culture + Molecular	10	33.33	[12,15,21,26,30,32,33,43,44,47]

n, number of included studies reporting each method; %, proportion of the 30 included studies reporting each method, calculated as (n/30) × 100. Categories are not mutually exclusive because individual studies could report more than one method.

**Table 6:** Ranges of spoilage bacterial counts and bacterial indicator groups in fresh cheeses.

Cheese Type	Microbial Group/Indicator	Count Range	References
Mozzarella	Total viable counts/mesophiles	7.7–9.33	[22,27,28,29]
	*Pseudomonas* spp.	2.78–8.04	[27,28,29,30]
	*Enterobacteriaceae*	2.9–8.04	[28]
	Coliforms	3–7.	[25,27,28,29]
	Lactic Acid Bacteria (LAB)	~6.6	[29]
Fior di latte/Fiordilatte	*Pseudomonas* spp.	6–7.5	[38,40,41,45]
	*Enterobacteriaceae*	~5–7.1	[38,40,41]
	Lactic Acid Bacteria (LAB)	~6.8	[45]
Stracciatella	*Pseudomonas* spp.	6–8.	[42]
	Coliforms/*Enterobacteriaceae*	5–8	[42]
Fresh cheese	Total viable counts	~6.5–8	[13,33]
	*Pseudomonas* spp.	6.9	[33]
	*Enterobacteriaceae*	5.8	[33]
	Lactic Acid Bacteria (LAB)	~6–6.7	[13,33]
Oaxaca/Panela/Cottage	Total viable counts	6.37–8.46	[12]
	Lactic Acid Bacteria (LAB)	8.25–8.31	[12]
	*Enterobacteriaceae*	3.05–6.71	[12]
Soft/semi-hard cheeses with blowing defects	Lactic Acid Bacteria (LAB)	6–8	[37]
	*Clostridium* spp./*Bacillus* spp.	5–6	[37]

Counts are expressed as log CFU/g. The symbol “~” indicates approximate values estimated from graphical data when exact numerical values were not reported in the text or tables of the original studies.

## Data Availability

The original contributions presented in this study are included in the article/Supplementary Materials. Further inquiries can be directed to the corresponding author.

## Supplementary Materials

The following supporting information can be downloaded at: https://www.mdpi.com/article/10.3390/microorganisms14091936/s1, PRISMA 2020 checklist; File S1—Optimized_Matrix_Q1 Journal.xlsx; File S2—Scoping Review Spoilage Microorganisms in Fresh Cow Cheese.xlsx. Reference [19] is cited in the Supplementary Materials.
